# MicroRNA-155 Hallmarks Promising Accuracy for the Diagnosis of Various Carcinomas: Results from a Meta-Analysis

**DOI:** 10.1155/2015/327287

**Published:** 2015-03-30

**Authors:** Chuancheng Wu, Qiuyan Liu, Baoying Liu

**Affiliations:** ^1^Department of Preventive Medicine, Fujian Province Key Lab of Environment and Health, Institution of Environment and Health, Major Subject of Environment and Health of Fujian Key Universities, School of Public Health, Fujian Medical University, No. 1 Xue Yuan Road, University Town, Fuzhou, Fujian 350108, China; ^2^School of Postgraduate Education, Fujian Medical University, No. 1 Xue Yuan Road, University Town, Fuzhou, Fujian 350108, China

## Abstract

*Background*. Recent studies have shown that microRNAs (miRNAs) have diagnostic values in various cancers. This meta-analysis seeks to summarize the global diagnostic role of miR-155 in patients with a variety of carcinomas. *Methods*. Eligible studies were retrieved by searching the online databases, and the bivariate meta-analysis model was employed to generate the summary receiver operator characteristic (SROC) curve. *Results*. A total of 17 studies dealing with various carcinomas were finally included. The results showed that single miR-155 testing allowed for the discrimination between cancer patients and healthy donors with a sensitivity of 0.82 (95% CI: 0.73–0.88) and specificity of 0.77 (95% CI: 0.70–0.83), corresponding to an area under curve (AUC) of 0.85, while a panel comprising expressions of miR-155 yielded a sensitivity of 0.76 (95% CI: 0.68–0.82) and specificity of 0.82 (95% CI: 0.77–0.86) in diagnosing cancers. The subgroup analysis displayed that serum miR-155 test harvested higher accuracy than plasma-based assay (the AUC, sensitivity, and specificity were, resp., 0.87 versus 0.73, 0.78 versus 0.74, and 0.77 versus 0.70). *Conclusions*. Our data suggest that single miR-155 profiling has a potential to be used as a screening test for various carcinomas, and parallel testing of miR-155 confers an improved specificity compared to single miR-155 analysis.

## 1. Introduction

Malignant tumor has become the number one killer to human health and early diagnosis of these malignancies remains a compelling challenge for the clinicians. For the serological diagnosis in cancer thus far, classical blood-based tumor markers like carcinoembryonic antigen (CEA), prostate specific antigen (PSA), and carbohydrate antigen (CA) have gained a lot of recognition in the diagnosis or prediction of variety malignant tumors. Nevertheless, the utility of these available tumor markers is limited by disappointing diagnostic accuracies, especially with respect to their applications in diagnosing early phase carcinomas or incapability in distinguishing aggressive tumors from the indolent ones [1, 2]. For instance, the accuracies of conventional CA15-3 and CEA revealed high volatilities for breast cancer detection, in which the discrepancy may due to ethnic group, sample size, or cancer subtype [3, 4]; even for PSA, which is widely used in routine clinical practice for the screening and management of prostate cancer patients, the accuracy is not satisfactory as well [5]. In this respect, development of suitable biomarkers is critical for diagnosing cancer or predicting patients' outcome.

Analysis of molecular genetic markers in biological fluids or tissues has been proposed as a useful tool for cancer diagnosis. MicroRNAs (miRNAs) are a class of short, single stranded, approximately 18–25 nucleotide noncoding RNAs and are cleaved from 70 to 100 nucleotide hairpin precursors by a complex protein system that involves the ribonucleases (RNases) III Drosha and Dicer, Pol-II-dependent transcription, and members of the argonaute family [6, 7]. Since their discovery, abnormal miRNA profiles have been reported to associate with diagnosis, prognosis, metastasis, and even survival in a variety of neoplastic diseases [8–11]. Among these various miRNAs, miR-155 which initially generated from an exon of a noncoding RNA transcribed from B cell integration cluster located on chromosome 21 is highlighted and widely studied. In cancers, miR-155 is shown as an oncogenic miRNA associated with hematological malignancies, lung cancer, breast cancer, and other carcinomas [11–16]. Interestingly, abnormal expression of miR-155 might represent a potential valuable marker for cancer detection [2, 12, 17–31]. However, the diagnostic accuracy of miR-155 for tumors was inconsistent or even contradictory in literature, which may be explained in part by the differences in study design, sample size, sample type, and race. Therefore, the present meta-analysis was conducted and estimated the pooled accuracy of miR-155 detection in diagnosing various carcinomas.

## 2. Materials and Methods

### 2.1. Search Strategy and Inclusion Criteria

The current meta-analysis followed the guidelines of the Preferred Reporting Items for Systematic Reviews and Meta-Analysis (PRISMA) Statement and methods [32]. The online PubMed, Embase, and Chinese National Knowledge Infrastructure (CNKI) databases were searched for all published articles without language restriction. The search terms were used as “cancer/carcinoma/tumor,” “microRNA-155/miR-155,” “circulating microRNA/miRNA,” and “diagnosis/sensitivity/specificity.” We manually searched the reference lists of eligible studies identified from the databases as well.

All eligible studies satisfying the following criteria were firstly included in our analysis: (1) miR-155 was assessed in cancer diagnostic studies; and (2) studies mentioned the sample number, sensitivity, specificity, AUC, and their 95% confidence intervals (CIs) or other more detailed information. Studies were excluded based on the following criteria: (1) studies that failed to explicitly state the control groups; and (2) review articles, meta-analysis, letters, commentaries, abstracts presented in conferences, and studies without complete data.

### 2.2. Data Extraction and Quality Assessment

Two reviewers independently evaluated the final set of selected articles. The extracted data elements of this study for diagnosis included the first author, year of publication, country of origin, number of patients, control sources, sample types, miRNA profiles, test method, diagnostic parameters, and other substantial information. In studies containing both a training and a validation group, data of each group was regarded as a single study in the meta-analysis. Any disagreement was resolved by consensus.

The evidence-based and critical review checklist of quality assessment of diagnostic accuracy studies (QUADAS) tool was used to assess each study's quality [33], and studies were evaluated as “Yes (low risk/high concern),” “No (high risk/low concern),” or “Unclear (unclear risk/unclear concern).” An answer of “Yes” will get a score of 1, while a “No” or “Unclear” answer gains a score of 0. Any disagreement was resolved by consensus.

### 2.3. Statistical Analysis

Statistical analysis was conducted utilizing Stata 12.0 (Stata Corporation, College Station, TX, USA) and Meta-disc 1.4 (XI Cochrane Colloquium, Barcelona, Spain) software. The bivariate meta-analysis model was employed to summarize the sensitivity, specificity, positive likelihood ratio (PLR), negative likelihood ratio (NLR), and diagnostic odds ratio (DOR) and generate the bivariate SROC curve with their corresponding 95% CIs. For the heterogeneity analysis, Spearman correlation coefficient was performed to analyze the threshold effect, while Cochran-*Q* test and *I* ^2^ test were used to assess heterogeneity from nonthreshold effect. *P* < 0.05 for Spearman correlation coefficient, or *P* < 0.01 for Cochran's *Q* test, or *I* ^2^ > 50%, all indicated an existence of significant heterogeneity [34]. The influence analysis and meta-regression were applied to trace potential sources of study heterogeneity. Deeks' funnel plot asymmetry test was employed to estimate the potential publication bias among studies, and *P* < 0.01 was considered to be representative of a significant statistical publication bias.

## 3. Results

### 3.1. Search Results

**Figure 1:**
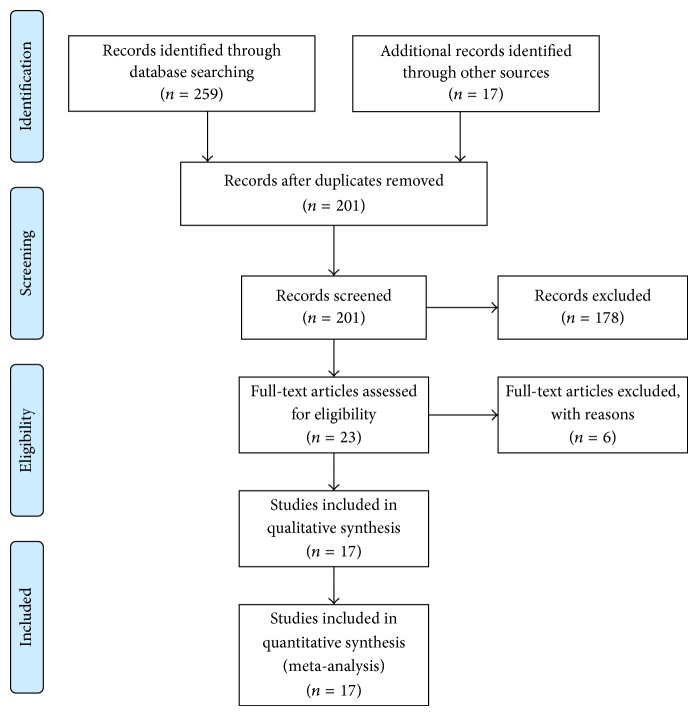
Flow diagram of the studies identification and selection.

Flow chart for study selection is shown in Figure 1. A total of 276 trials were identified by our electronic database search (*n* = 259) and through other sources (*n* = 17). After the duplicates were removed, 201 unique abstracts remained. The titles, abstracts, and key words were then carefully evaluated, and 178 studies were excluded due to the status of review articles, letters, basic research, and so forth. The retrieved 23 studies were conducted for more detailed evaluation, and 6 of them were further excluded due to the lack of sufficient data and were all discarded. Therefore, only 17 publications [2, 12, 17–31] seemed to meet all of the inclusion criteria and none of the exclusion criteria.

### 3.2. Study Characteristics and Quality Assessments

In this meta-analysis, the final set of 17 diagnostic studies included a total of 886 patients with various cancers and 670 healthy control individuals. The sample size of cancer patients in each study varied from 20 to 103, and control numbers varied from 6 to 92. All the cancer patients were diagnosed based on the histopathological examination. Among the 17 studies, 6 studies had an ethnicity of Caucasian, 11 studies had an ethnicity of Asian, and 13 studies conducted single miR-155 assay for cancer detection. Besides, all the 17 studies performed reverse transcription quantitative PCR (RT-qPCR) for the miRNAs detection, and the specimen type included serum [12, 17–20, 23, 24, 30], plasma [21, 22, 25, 26, 31], whole blood [2, 29], and tissue [27, 28]. The main features of the included studies were described in Table 1.

**Figure 2:**
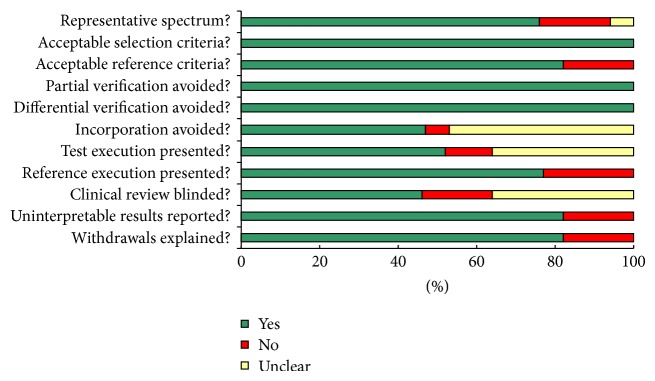
Summary of assessment of the included studies analyzed using the quality assessment for studies of diagnostic accuracy (QUADAS) tool: proportion of studies with low (Yes), mediate (Unclear), and high risk of bias (No).

We estimated the quality of the 17 included publications according to the 14-item QUADAS assessment tool [33]. Twelve of the 17 studies had QUADAS scores more than 10 and revealed lower risks of bias, suggesting a high quality of the included investigations (Table 1 and Figure 2).

### 3.3. Heterogeneity

As displayed in Table 2, the *P* values of Spearman correlation coefficient in the single miR-155 assay were less than 0.05, indicating the existence of heterogeneity from threshold effect in analysis. Moreover, except the Caucasian-based miR-155 test, heterogeneity generated by nonthreshold effect appeared in all the other pooled analyses, in which the *P* values of Cochran's* Q* test were all less than 0.1, accompanied by *I* ^2^ > 50%.

### 3.4. Diagnostic Performance

**Figure 3:**
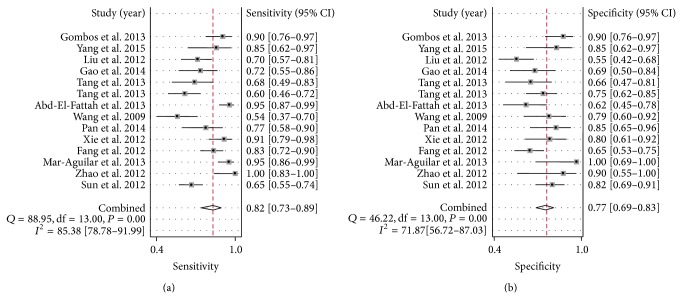
Forest plots of the pooled sensitivity and specificity for single miR-155 in detecting cancers. (a) Sensitivity; (b) specificity. Only the first author of each study is given. Sensitivity and specificity were given with confidence intervals (CIs).

**Figure 4:**
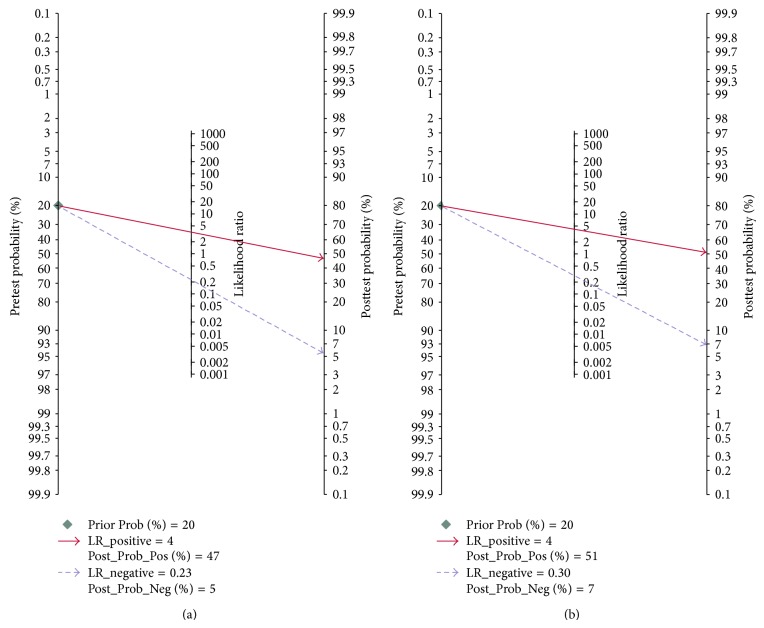
Pre- and posttest probabilities of single and paneled miR-155 analyses. (a) Single miR-155 test; (b) paneled miR-155 test.

**Figure 5:**
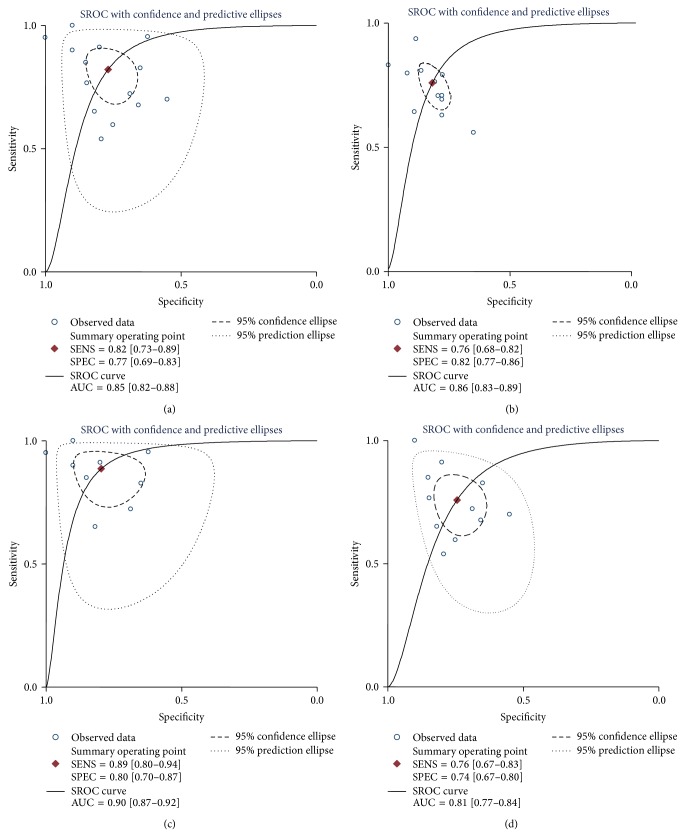
The SROC curves of the pooled individual analyses. Sample size is indicated by the size of the square. The regression SROC curve indicates the overall diagnostic accuracy. (a) Single miR-155 test; (b) paneled miR-155 test; (c) serum-based miR-155 test; (d) Asian population-based miR-155 test. AUC: area under curve,* Q*: index; SE: standard error; SROC: summary receiver operator curve.

Since there existed significant heterogeneity between studies, the random-effects model was applied in the meta-analysis. Forest plots of the sensitivity and specificity for miR-155 validation in diagnosing cancers are shown in Table 3 and Figure 3. The pooled results of sensitivity, specificity, PLR, NLR, and DOR for single miR-155 test were 0.82 (95% CI: 0.73–0.88), 0.77 (95% CI: 0.70–0.83), 3.56 (95% CI: 2.55–4.95), 0.23 (95% CI: 0.14–0.37), and 15.34 (95% CI: 7.32–32.17), respectively. For the parallel testing of miR-155, the results conferred a better performance for some parameters: the specificity was 0.82 (0.77–0.86), and PLR was 4.23 (3.12–5.75), corresponding to an AUC of 0.86. Furthermore, the posttest probability was calculated, and single miR-155 harbored a pretest probability of 20% and a posttest probability of 47%, while the paneled miR-155 test achieved a pretest probability of 20% and a posttest probability of 51% (Figure 4). The SROC curve for single and paneled miR-155 validations was displayed in Figures 5(a)-5(b).

### 3.5. Subgroup Analyses

The results of our subgroup analyses are summarized in Table 3 and Figures 5(c)-5(d). A comparison of miRNA expression patterns in serum and plasma showed that the sensitivity (0.78 versus 0.74), specificity (0.77 versus 0.70), DOR (15.61 versus 5.54), PLR (3.47 versus 2.02), and AUC (0.87 versus 0.73) were higher in serum-based assay than in plasma, providing additional evidences for the use of serum miR-155 as relatively reliable matrix in diagnosing carcinomas. We further performed an analysis based on ethnicity. The data exhibited that Caucasian population-based study yielded a combined sensitivity of 0.94 (95% CI: 0.89–0.97) and specificity of 0.79 (95% CI: 0.69–0.87) under the SROC curve, accompanied by DOR of 61.93 (23.00–166.75), PLR of 5.79 (1.51–22.24), NLR of 0.08 (0.04–0.15), and AUC of 0.96; for the Asian population-based assay, the pooled sensitivity, specificity, DOR, PLR, NLR, and AUC were 0.72 (95% CI: 0.68–0.76), 0.72 (95% CI: 0.68–0.76), 8.03 (95% CI: 4.82–13.38), 2.68 (95% CI: 2.07–3.46), 0.38 (95% CI: 0.29–0.50), and 0.81, respectively. Therefore, a difference in diagnostic accuracy was displayed between these two ethnicities, with a better diagnosis accuracy of miR-155 found in Caucasian populations.

### 3.6. Influence Analysis and Metaregression

**Figure 6:**
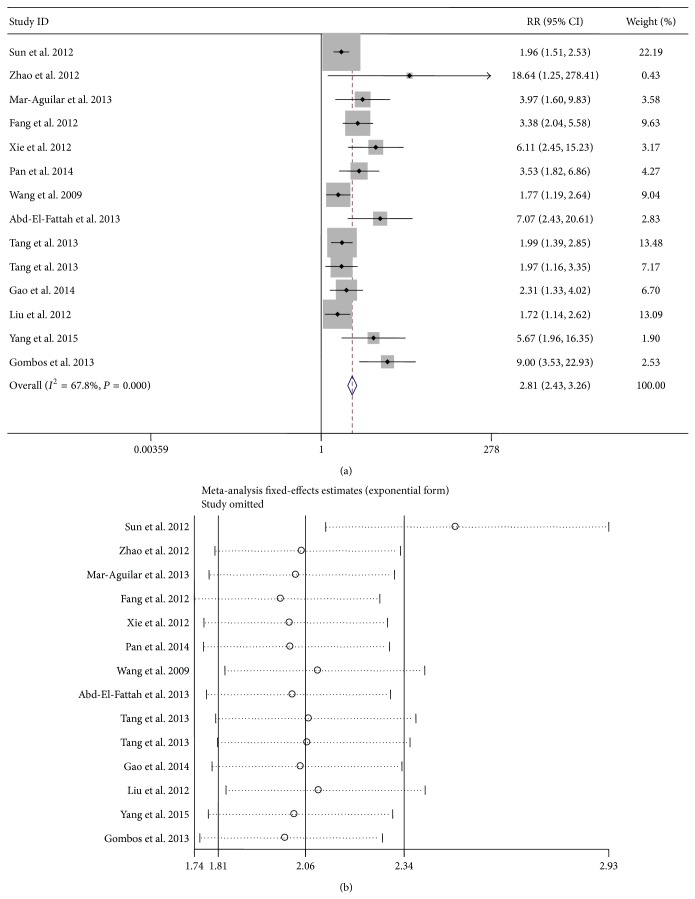
Influence and outlier detection analyses of the overall pooled study: the intermediate variable of RR (a) and outlier detection analysis (b). RR: relative risk.

As shown in Figure 6, the influence analysis and outlier detection identified one outlier study [17]. After excluding the outlier, the overall pooled sensitivity for single miR-155 increased from 0.82 to 0.84, specificity decreased from 0.77 to 0.76, DOR increased from 15.34 to 16.41, PLR decreased from 3.56 to 3.54, NLR decreased from 0.23 to 0.21, and AUC increased from 0.85 to 0.86. Moreover, the *I* ^2^ for sensitivity declined from 85.38 to 85.0%, suggesting that the outlier study is likely a source of heterogeneity; however, the *P* value of Cochran's *Q* test as well as *I* ^2^ for the pooled study altered unconspicuously, hinting that substantial heterogeneity from nonthreshold effect exists among studies.

Therefore, we further conducted meta-regression analysis by adding a total of 7 prespecified covariates (design type, study quality, specimen type, ethnicity, cut-off value setting, number of cases, and number of controls) to the bivariate model to assess their impacts on sensitivity and specificity. In consideration of the small study size of our analysis, a permute metaregression module was employed with a check value of 10,000, and each time only two covariates were estimated by Stata 12.0 software. Our data exhibited that ethnicity (*P* = 0.037) and cut-off value setting (*P* = 0.049) might introduce significant heterogeneity in both sensitivity and specificity, while design type (*P* = 0.712), sample type (*P* = 0.490), study quality (*P* = 0.311), number of cases (*P* = 0.069), and number of controls (*P* = 0.081) showed low likelihood of sources of interstudy heterogeneity (not shown).

### 3.7. Publication Bias

**Figure 7:**
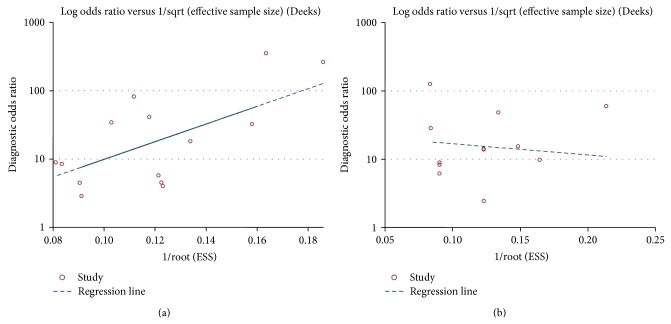
Funnel plot test for the assessment of potential bias for the analyses. (a) Deeks' funnel plot asymmetry test for the single miR-155 analysis, *P* = 0.124; (b) Deeks' funnel plot asymmetry test for paneled miR-155 analysis, *P* = 0.767. A *P* value less than 0.1 was considered to be representative of a significant statistical publication bias.

Publication bias of the included studies was checked by Deeks' funnel plot asymmetry test. The slope coefficient was associated with a *P* value of 0.124 and 0.767, respectively, for the single and paneled miR-155 analysis, suggesting an existing low likelihood of publication bias (Figure 7). For the subgroup analysis, the slope coefficient presented *P* values of 0.386 and 0.151, respectively, for the matrix and ethnicity-based assays, also showing that no obvious publication bias existed (data not shown).

## 4. Discussion

Sensitive and specific tumor biomarkers are essential to early cancer detection and diagnosis and for undertaking novel therapeutic trials and prevention strategies in clinic. In recent years, aberrant expression of miRNAs has been widely reported in various carcinomas [2, 8–31]. Among all uncovered miRNAs, miR-155 is no doubt one of the most attractive ones, which is reported to have potential diagnostic and prognostic value for cancers [2, 12, 17–31]. As the diagnostic role of miR-155 has not yet been well elucidated thus far, we performed a comprehensive meta-analysis and estimated the pooled accuracy of miRNA-155 for cancer detection.

Our data showed promising accuracy for single miR-155 detection in diagnosing tumors, in which the overall pooled sensitivity was 0.82 and specificity was 0.77, with an AUC of 0.85, suggesting that miR-155 achieved a relatively high accuracy for cancer detection. In a meta-analysis study, the diagnostic odds ratio (DOR), defined as the ratio of the odds of a true positive to the odds of a false-positive, is an important indicator of diagnostic accuracy: a DOR value less than 1.0 often indicates a low discriminating ability in the diagnostic test [35, 36]. In our data, the pooled DOR was presented as 15.34, indicating a better discriminatory performance of miR-155 for cancers. Moreover, we determined the pooled PLR and NLR to obtain a more comprehensive view of their diagnostic accuracy: the PLR of 3.56 suggested that patients with tumors had nearly fourfold higher chance of being miR-155 test positive than other healthy donors. Meanwhile, the pooled NLR was found to be 0.23, implying that, in a negative result from the miR-155 test, only 23% is likely to be false-negative. The published studies had addressed the use of miR-155 expression as biomarker for cancer detection, in which the diagnostic accuracy of miR-155 was highlighted [18–20, 27, 28]. Recently, a newly published meta-analysis containing three studies for miR-155 showed that miR-155 has the potential diagnostic value for breast cancer detection, with a pooled sensitivity of 0.79 and specificity of 0.85 [37].

Interestingly, the parallel testing of miR-155 seemed to achieve a high diagnostic accuracy for the differentiation between cancer patients and healthy people, with a sensitivity of 0.76 and specificity of 0.82, displaying an AUC of 0.86. Strikingly, the pooled specificity was presented as 0.82, indicating a more specific discriminatory performance of miR-155 panel than single miR-155 test in cancers. Moreover, the pooled PLR of 4.23 also suggested an increased diagnostic performance for the combined miR-155 test. Research from Gombos et al. [28] demonstrated that a combination of three circulating miRNAs, including miR-155, further enhanced the discriminative power of the test for oral squamous cell carcinomas. Tang et al. [25] recently presented the similar results as well. Nevertheless, as shown in our data, the pooled sensitivity, DOR, and NLR of the paneled miR-155 test were discounted when compared to the single miR-155 assay; therefore, more data are still needed to confirm the real diagnostic signature of parallel testing of miR-155 for cancers.

Furthermore, we conducted subgroup analyses based on the following variables like sample type and ethnicity. Notably, our analysis based on sample type showed that using serum miR-155 as biomarker for detecting cancers yielded an overall sensitivity of 0.78 and an overall specificity of 0.77. The AUC of 0.87 and DOR of 15.61 also indicated a relatively high level of the diagnostic accuracy. Data from a newly published meta-analysis revealed that serum-based miRNA assay seemed to undergo a higher combined DOR, NLR, and AUC than that of plasma-based test, suggesting that serum may be a better matrix for diagnostic profiling of miRNAs in breast cancer [35]. Similarly, a published article also observed a diagnostic difference between serum and plasma miRNA assays in hematologic cancers [38]. Evidence from Wang et al. [39] demonstrated that the coagulation process may affect the spectrum of extracellular miRNAs in the blood, implying that different matrices may harvest different diagnostic accuracies for miRNA detection. On the other hand, in terms of ethnicity-based miR-155 tests, the Caucasian group yielded a pooled sensitivity of 0.94, specificity of 0.79, and AUC of 0.96, displaying the highest diagnostic accuracy in this meta-analysis. Researches have shown that different racial expression profiles are associated with circulating miRNA concentrations, hinting that miRNA signature varies among ethnicity [40, 41]. Additionally, result from a meta-analysis study also suggests that miRNA profiling assay may be more precise in Caucasian populations [35]. Consistent with these data, our results showed promising accuracy for Caucasian-based miR-155 validation in diagnosing cancers. Notwithstanding, the results of Caucasian-based analysis were pooled from 3 studies only, for which the small study size compromised the accuracy of the data, although no heterogeneity was found among these studies.

In this meta-analysis, heterogeneity from threshold effect existed in the single miR-155 test. The threshold effect was mainly generated by the different cut-off value setting or thresholds used in different studies. The cut-off values for miR-155 test were not uniformed among studies, which may further contribute the heterogeneity from threshold effect. On the other hand, the pooled DOR is often used to discuss the heterogeneity caused by nonthreshold effects [35]. We found that the DOR of each study (except the Caucasian-based analysis) did not distribute along a straight line with the pooled DOR in the forest plots, and the *P* values in Cochran's *Q* test were all less than 0.01, accompanied by *I* ^2^ more than 50%, also indicating substantial heterogeneity from nonthreshold effect in studies. For its causes, the different measurement methods or sample types may contribute to heterogeneity sources. In our study, although the detecting methods for miR-155 were all based on RT-qPCR, the ethnicities and sample type were different among studies. Moreover, the participants enrolled in the tests were not unified for their disease stages, conditions, or other concomitant diseases. Therefore, we further conducted influence and metaregression analyses to assess the contribution of the factors above and found that the outlier studies as well as different cut-off values used among studies were the sources of heterogeneity.

Our data demonstrated that miR-155 has a potential of being promising biomarker of cancers. However, several points should be addressed in their interpretation. First, for the researchers, how to select an appropriate cut-off value for the test is a vital point. All the enrolled studies in this meta-analysis varied for their cut-off values setting, and most of the studies used median or mean value in their laboratory or hospital as the cut-off value thus far. Second, as we obtained different diagnostic accuracy for the matrix-based studies, which matrix should be used for the test, plasma, serum, whole blood, tissue, or other bodily substances? Serum, plasma, and blood are easy to obtain and are convenient to be monitored routinely, whereas the tissues are widely utilized resources for miRNA study currently, especially in some research laboratories. Last, other than single miR-155 test, the miR-155 panel similarly revealed promising accuracy for the cancer detection, so which should be conducted for the diagnosis test, single or paneled miR-155 test, still warrants further investigations. Last, difficulty still remains for miR-155 as a new diagnostic marker for various carcinomas: aberrant miR-155 signature was depictured in various cancers instead of a particular one, which has compromised the diagnostic specificity when used in the practice. In this aspect, the combination of miR-155 with the circulating protein-biomarkers may be a novel potential tool for cancer detection. A new proof has shown that parallel testing of miR-155 and serum CEA level preoperatively can afford more accurate information for colon cancer diagnosis [42].

In summary, our findings clearly demonstrated that miR-155 confers high diagnostic accuracy for cancer detection, and combined sequential testing of miR-155 achieves an improved specificity compared to single miR-155 assay. Despite the promising results, the current study does have limitations involving the small study size as well as the substantial heterogeneity from nonthreshold effect existing among studies. In consequence, the combined diagnostic indices of miR-155 in this study are unable to completely mirror its actual diagnostic value for cancers, and, further, large cohort studies are still warranted.

## Figures and Tables

**Table 1:** The main features of the included studies for miR-155 in the diagnosis of cancers. DLBCL: diffuse large B cell lymphoma; AML: acute myeloid leukemia; QUADAS: quality assessment for studies of diagnostic accuracy.

Author	Year	Ethnicity	Patients (control)	Cancer type	Sample	Test method	MicroRNA profiling	QUADAS scores
Mar-Aguilar et al. [18]	2013	Caucasian	61 (10)	Breast cancer	Serum	RT-qPCR	miR-155	12

Sun et al. [17]	2012	Asian	103 (55)	Breast cancer	Serum	RT-qPCR	miR-155	13

Zhao et al. [19]	2012	Asian	20 (10)	Breast cancer	Serum	RT-qPCR	miR-155	12

Fang et al. [12]	2012	Asian	75 (77)	DLBCL	Serum	RT-qPCR	miR-155	12

Xie et al. [20]	2012	Asian	45 (30)	AML	Serum	RT-qPCR	miR-155	8

Pan et al. [21]	2014	Asian	30 (26)	Pancreatic cancer	Plasma	RT-qPCR	miR-155	8

Wang et al. [22]	2009	Asian	30 (29)	Pancreatic cancer	Plasma	RT-qPCR	miR-155	12

Abd-El-Fattah et al. [23]	2013	Caucasian	65 (37)	Lung cancer	Serum	RT-qPCR	miR-155	11

Gao et al. [24]	2014	Asian	36 (32)	Lung cancer	Serum	RT-qPCR	miR-155	9

Tang et al. [25]	2013	Asian	96 (92)	Lung cancer	Plasma	RT-qPCR	Single miR-155 and paneled miRNAs	12

Liu et al. [26]	2012	Asian	60 (60)	Esophageal cancer	Plasma	RT-qPCR	miR-155	9

Yang et al. [27]	2015	Asian	20 (20)	Rectal cancer	Tissue	RT-qPCR	miR-155	12

Gombos et al. [28]	2013	Caucasian	40 (40)	Oral squamous cell cancer	Tissue	RT-qPCR	miR-155	9

Häusler et al. [29]	2010	Caucasian	24 (15)	Ovarian cancer	Whole blood	RT-qPCR	More than thirty paneled miRNAs involved miR-155	11

Heneghan et al. [2]	2010	Caucasian	83 (63)	Breast cancer	Whole blood	RT-qPCR	Three paneled miRNAs involved miR-155	12

Roa et al. [30]	2012	Caucasian	24 (6)	Lung cancer	Sputum	RT-qPCR	Five paneled miRNAs involved miR-155	12

Zheng et al. [31]	2011	Asian	74 (68)	Lung cancer	Plasma	RT-qPCR	Three paneled miRNAs involved miR-155	12

**Table 2:** Heterogeneity assessment of the individual pooled analysis.

Analyses	Spearman correlation coefficient	Cochran's *Q* test	*I* ^2^ test (%)	Heterogeneity	
				Threshold effect	Nonthreshold effect
Single miR-155	−0.363^a^ *P* = 0.203	46.73^b^ *P* = 0.0000	72.2	No	Yes
Panel miR-155	−0.606^a^ *P* = 0.037	35.98^b^ *P* = 0.0002	69.4	Yes	Yes
Serum-based	−0.571^a^ *P* = 0.180	16.06^b^ *P* = 0.0134	62.6	No	Yes
Plasma-based	0.359^a^ *P* = 0.553	10.42^b^ *P* = 0.0340	61.6	No	Yes
Asian	−0.409^a^ *P* = 0.212	25.23^b^ *P* = 0.0049	60.4	No	Yes
Caucasian	0.500^a^ *P* = 0.667	2.16^b^ *P* = 0.3394	7.4	No	No
Overall	−0.385^a^ *P* = 0.052	85.38^b^ *P* = 0.0000	70.7	No	Yes
Outliers excluded	−0.418^a^ *P* = 0.156	46.76^b^ *P* = 0.0000	70.3	No	Yes

^a^The value of sensitivity and (1 − specificity); ^b^ *Q* value.

**Table 3:** Summary table of the diagnostic accuracy of miR-155 for various cancers.

Analyses	Sensitivity (95% CI)	Specificity (95% CI)	DOR (95% CI)	Positive LR (95% CI)	Negative LR (95% CI)	AUC (95% CI)
MicroRNA profile						
Single miR-155	0.82 (0.73–0.88)	0.77 (0.70–0.83)	15.34 (7.32–32.17)	3.56 (2.55–4.95)	0.23 (0.14–0.37)	0.85
Paneled miR-155	0.76 (0.68–0.82)	0.82 (0.77–0.86)	14.27 (7.98–25.53)	4.23 (3.12–5.75)	0.30 (0.22–0.40)	0.86
Sample types						
Plasma-based	0.74 (0.58–0.71)	0.70 (0.63–0.76)	5.54 (2.79–11.03)	2.02 (1.65–2.48)	0.41 (0.27–0.63)	0.73
Serum-based	0.78 (0.74–0.82)	0.77 (0.71–0.82)	15.61 (7.03–34.67)	3.47 (2.40–5.02)	0.23 (0.13–0.41)	0.87
Ethnicity						
Asian	0.72 (0.68–0.76)	0.72 (0.68–0.76)	8.03 (4.82–13.38)	2.68 (2.07–3.46)	0.38 (0.29–0.50)	0.81
Caucasian	0.94 (0.89–0.97)	0.79 (0.69–0.87)	61.93 (23.00–166.75)	5.79 (1.51–22.24)	0.08 (0.04–0.15)	0.96
Overall	0.79 (0.73–0.84)	0.79 (0.75–0.84)	14.62 (9.10–23.48)	3.86 (3.05–4.90)	0.26 (0.20–0.35)	0.86
Outliers excluded	0.84 (0.74–0.90)	0.76 (0.68–0.83)	16.41 (3.32–36.77)	3.54 (2.49–5.05)	0.21 (0.13–0.36)	0.86

CI: confidence interval; LR: likelihood ratio; DOR: diagnostic odds ratio; AUC: area under the curve.
